# Applicability of an Immersive Virtual Reality Exercise Training System for Office Workers during Working Hours

**DOI:** 10.3390/sports10070104

**Published:** 2022-06-29

**Authors:** Evlalia Touloudi, Mary Hassandra, Evangelos Galanis, Marios Goudas, Yannis Theodorakis

**Affiliations:** School of Physical Education, Sport Science and Dietetics, Department of Physical Education and Sport Science, University of Thessaly, 42100 Trikala, Greece; maryhassandra@gmail.com (M.H.); egalanis@uth.gr (E.G.); mgoudas@pe.uth.gr (M.G.); theodorakis@uth.gr (Y.T.)

**Keywords:** VR exercise system, office workers, sedentary, acceptability, usability, exercise

## Abstract

Virtual reality is a computer-generated simulation of a real or imaginary three-dimensional environment that has entered our lives, particularly for gaming. Lately, it has been permeating into many aspects of our everyday life, such as exercise. It is important to ascertain whether exercise in an immersive virtual reality environment can be accepted from employees and lead to positive outcomes for them. The aim of this exploratory study was to examine the acceptance, future adoption, interest/enjoyment and usability of an immersive virtual reality system for exercise training by office workers during breaks within their working hours. A total of 40 female employees participated in the study with a mean age of 42.58 years (SD 10.77). Participants were requested to complete two sequential 15-min dual task cycling sessions corresponding to two experimental conditions. The first, condition A, involved cycling in a virtual environment, wearing a virtual reality head mounted display, and responding to cognitive tasks by answering multiple choice questions—on a screen, using a joystick. The second, condition B, involved cycling on a static bicycle and simultaneously responding to cognitive tasks by answering multiple choice questions in a real environment. After completion of the two conditions, participants responded to a series of scales regarding each of the experimental conditions and to a semi-structured interview. The results showed that participants noted a significant preference for the immersive virtual reality exercise, condition A, compared to condition B (bike only); and their acceptance, interest/enjoyment, usability and intention for future use were high. The qualitative data showed increased intention for future use, feelings of control and presence and most of the participants did not encounter any difficulties or require extra help to understand the immersive virtual reality system. Overall, exercising during working hours with an immersive virtual reality exercise system was well perceived by office workers and applicable. However, the effects of the immersive virtual reality training system on physical and mental health and the employees’ adherence to the exercise program should be tested with a longer intervention program.

## 1. Introduction

According to the World Health Organization (WHO), a great percentage of adults do not regularly meet the recommendations of physical activity needed to maintain or improve their health. This has as a result of poor health outcomes such as all-cause mortality, cardiovascular disease and type-2 diabetes, but also poor mental health and low general wellbeing. A strong recommendation is to limit the amount of sedentary time by replacing it with any intensity of physical activity [1]. The American College of Sports Medicine (ACSM) recommendation about physical activity is the daily goal of 30 min or more of moderate-intensity exercise. To attain the daily goal, individuals can have multiple 10-min or more bouts of exercise [2]. Many studies have examined the effects of short bouts of exercise versus one continuous bout per day. According to the results, multiple short sessions may enhance adherence to the exercise program, help weight loss and show similar improvements in cardiorespiratory function compared with long sessions [3]. In sedentary adults, multiple 10-min sessions of exercise may have positive effects in fitness and health benefits [4].

Specifically, according to a WHO recommendation, it is crucial for the health of sedentary employees to take a break from their job and exercise for 10–15 min. Regular exercise can lead to positive effects not only for the overall health of employees, but also to help them be more productive and decrease absenteeism. Employees can include multiple 10-min or more exercise bouts in their daily program to meet the WHO recommendations (>30 min per day) [1].

Currently, increased computer use has replaced manual work to a large extent [5]. The development of technology has offered new opportunities to all employees, including the disabled ones, for all stages of their lives and careers. Moreover, the advanced technology and remote work has reduced the risk of dangerous tasks, but led to difficulties in the determination of boundaries between private life and work, a lack of communication and a general psychosocial risk [6]. Additionally, employees face increased demands for performing faster and more complex tasks. A great percentage of employees report musculoskeletal disorders as the most serious work-related problem [5]. The costs of work-related illnesses are high, compared with the cost of wellbeing; therefore, mental and physical health-promoting programs are encouraged [6].

Many studies have been focusing on the efficacy of exercise in the workplace to improve employees’ physical health, fitness, musculoskeletal problems, their productivity and mental health. Evidence shows that both endurance and muscle strengthening exercise can improve employees’ health parameters such as body composition, motor, musculoskeletal and cardiorespiratory fitness and prevent health issues [7]. Undeniably, exercise in the workplace, especially for white-collar workers, can be beneficial for many parameters of their physical health, such as their fitness level, functional capacity, maximal strength, fat-free mass and musculoskeletal problems. This is very important because musculoskeletal pain has a negative effect on productivity and increases absenteeism [8]. Simultaneously, evidence shows that mental health can be improved because individuals have shown satisfaction towards their participation in training programs, improved their personal relationships [9] and increased perception about their health [10]. Additionally, implementing exercise programs in the workplace can lead to short-term financial savings by reducing healthcare costs and increasing the productivity of employees [11]. Work-related factors such as absenteeism, workability and work performance can also show positive effects from the employees’ participation to exercise programs [12].

Immersive virtual reality (IVR) is a computer-generated simulation of a real or imaginary three-dimensional environment using a head mounted display and controllers connected with an application. According to researchers, individuals can exercise through the VR system and respond realistically to the virtual stimuli just like in real life [13]. The VR training is considered appropriate and can lead to positive effects in physiological, psychological and rehabilitation factors compared to conventional exercise [14]. Additionally, it is a feasible way of exercising and a powerful asset to increase physical and cognitive activity in both young and old individuals, since the elderly have reported similar experiences with younger adults [15]. So, a VR exercise program can be used for different ages, not only for aerobic, but also for muscle strengthening activities [16].

Recently, IVR has been widely used for many reasons, such as rehabilitation, training and prevention. For instance, exercise in a virtual environment has been used to help obese children to reach health recommendations [17], and healthy adults to improve balance and decrease the risk of falls [18]. Additionally, training in a virtual reality environment has been used by coaches for their athletes, especially in team ball sports, to fit them properly for any situation in the field [19].

A recent survey supports that since exercise is considered to have a therapeutic effect in chronic diseases, a combination of IVR and exercise can enhance the therapeutic results [20]. At the same time, IVR exercise programs have a positive effect on cognitive ability, attention and memory but also on non-cognitive factors such as depression and anxiety [21]. The combined exercise with cognitive training in an IVR environment has increasingly been studied, especially in patients with mild cognitive impairment symptoms. Hassandra et al. 2021 [22] employed an IVR system including physical and cognitive training for exercise patients with mild cognitive impairment. Their results showed that the system was acceptable, usable and tolerable for patients and that it could be used to promote their physical and cognitive health. Moreover, evidence shows that IVR exercise can lead to positive effects for mental health, particularly on depression and anxiety symptoms [23].

However, except for the positive effects of IVR exercise, it is important to take into consideration the adverse effects of IVR. Many people feel dizziness, discomfort or nausea while wearing the IVR headset and participating in IVR experiences. This situation is called cybersickness or simulator sickness [24].

Combined motor and cognitive dual-task is very common in our everyday life. According to MacPherson (2018) “dual-tasking is the ability to perform two tasks simultaneously and measures a component of executive function as participants are required to coordinate their attention to both tasks while they are being performed” [25]. According to studies, dual-task situations can improve diagnosis, prevention and management of cognitive impairment and falls [26]. Specifically, evidence shows that movement and posture include motor and cognitive components. According to Yogev-Seligmann et al. 2008 [27] during dual-tasking, one or both tasks are deteriorated, except for the situation when the cognitive demand is very low. However, if the motor skill is automated, the simultaneous execution of another task may not affect the task performance. In older adults, when simple tasks are less automatic, dual task costs may be increased. Hence, dual-task can become a useful clinical marker of cognitive impairment and fall risk, because it can worsen cognitive or motor disfunctions [26].

Virtual reality systems have recently started to be used in workplace environments for workers’ stress reduction, relaxation, wellbeing improvement, work safety, work-related duties and thoughts distraction and have shown great expectation and support for VR interventions [28,29,30]. However, we have not detected a study that compared the perceptions of office workers regarding an IVR exercise system versus a static bicycle during working hours for training during working time. Therefore, the aim of this study was to examine the applicability of an IVR exercise system (compared to condition B: bike only) that allows office workers to exercise during their break within working hours. More specifically, we explored the employees’ acceptance, future adoption, interest/enjoyment and usability of the IVR training system, because it can be used as a motivational tool for them to be more physically active. We expect that participants will prefer and enjoy the IVR training condition compared to the bike only condition. The novelties of this study are new data for the acceptance, usability, future adoption and interest/enjoyment of office workers towards the IVR training system during their working hours.

## 2. Materials and Methods

### 2.1. Participants and Setting

A total of 40 female office workers participated in this study. They ranged in age from 20 to 61 years old, with mean age 42.58 years (SD 10.77). Their educational level, habits of physical activity and technology use are presented in Table 1. The setting was administration offices in a public sector organization, in Volos, a city in central Greece. The administration offices employ 202 office workers, with 65 men and 137 women.

### 2.2. Ethics Approval and Consent to Participate

Permission for the study was granted by the institution’s ethics committee (approval number: 1829, 13 October 2021). The confidentiality of personal data will be assured with regulation (EU) 2016/679 (general data protection regulation). The participants were informed orally for the procedure, provided written information and signed the consent form. Additionally, they were encouraged to ask questions and received complete and detailed answers.

### 2.3. IVR System—Meta Quest (Devices and Application)

**Figure 1:**
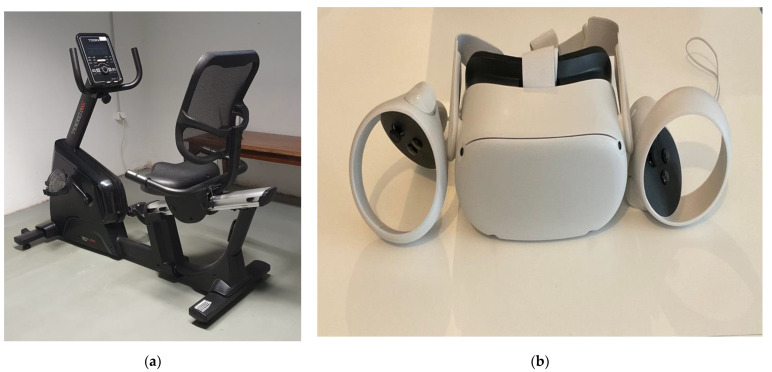
(**a**) The cycle-ergometer; (**b**) the Meta Quest 2 head mounted display (HMD) and controllers.

**Figure 2:**
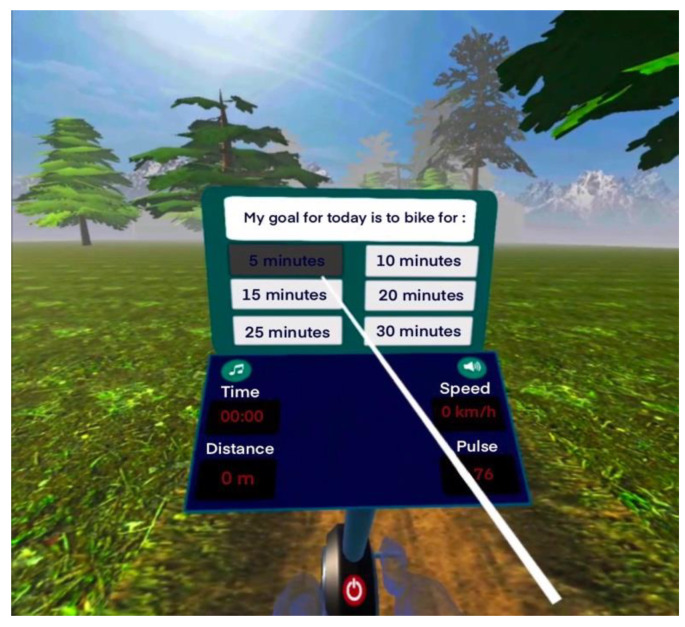
Selection of exercise duration.

A cycle-ergometer (stationary seated bike type; Toorx, Chrono Line, BRX R 300) with Bluetooth capability has been used for the measurements, as an ideal solution for the participants to control their training conditions and minimize the risk of falls. The cycle-ergometer was connected with the IVR application, which includes motivational techniques to address the issue of low motivation to exercise and the IVR head mounted display and controllers. For the IVR projection, the Meta Quest 2 (Fcebook technologies, LCC, Hacker Way, Menlo Park, CA, USA) device was used (Figure 1 and Figure 2). For example, participants could choose their exercise goal (exercise duration), have feedback for their training performance (cycling time, total distance, correct answers on cognitive exercises), task crafting (selection of music to enjoy while practicing) and self-monitoring (screen shows distance, time and speed data). The participants had the opportunity to choose the landscape in which they would like to practice (forest, beach, snowy landscape). In this study, all participants performed in the forest. The IVR system was developed by ORAMA-VR and Biomechanical Solutions Engineering, on the basis of interviews with older people with mild cognitive impairment.

In the present study, the IVR training system was selected to be used by sedentary workers, as an exercise option during working hours. One of the reasons for the selection of the IVR bike is that it is a safe and attractive way to exercise in the workplace during the work break. Additionally, it is not time consuming, employees can choose their exercise duration by themselves and the cost is lower compared with a gym room in the workplace. Thus, in this way employees can take advantage of their work break to exercise.

The IVR training system used is called VRADA (VR exercise App for Dementia and Alzheimer’s patients), Version 4.1. This IVR system (Version 3.7) was used with university students and patients with mild cognitive impairment as a dual task training aimed to promote their physical and cognitive health and assess its acceptability, tolerability and usability [22]. The results of this study were very encouraging, so it could be interesting to assess its applicability in different populations. The VRADA system has advantages that can meet the needs of employees, such as lack of time to exercise. Additionally, it is a very pleasant and attractive way to exercise and it might also be applicable to the employees, to have short breaks from work to exercise in their workplace, in order to maintain their physical health and avoid the negative effects from their sedentary work. (Supplementary Material S1—Video demo)

Firstly, the user must select the landscape for their training and then their cycling goal (training duration) within the IVR. As a selection mechanism, an IVR controller was used with a raycast allowing the user to choose an answer by pointing the ray at the button and pressing the trigger button on the controller.

Afterwards, they selected which word or phrase they would like to hear and repeat during their performance, such as “calmly”, “I can”, “I will do well”, “Very nice” or no words. Then, the participants could begin their performance on the cycling-ergometer. They also had the opportunity to change the song they were listening to, from a list of preloaded tracks. At the end of the cycling program, they received feedback from the application about the correct and incorrect answers from the cognitive tasks and they could evaluate their experience through questions, e.g., “Are you tired today?”, “Did you like the way you exercised today?”, “How many animals did you see?”, “How often did you repeat the word or phrase during the exercise?”. In this study, cognitive tasks were used as a way of distraction to avoid possible boredom during cycling. There was not a suggestion to the participants to focus on the cognitive tasks.

### 2.4. Measures

#### 2.4.1. Personal Innovativeness

A personal innovativeness questionnaire was used to find out users’ beliefs about technology adoption decisions, with a Cronbach’s α 0.77 [31]. The questionnaire included 4 items e.g., “I am the kind of person who looks forward to experimenting with new technologies” and participants were requested to answer through a 5-point Likert scale ranging from 1 (strongly disagree) to 5 (strongly agree).

#### 2.4.2. Acceptance

Three factors were used to assess participants’ acceptance for the VRADA app: 1. Perceived enjoyment; 2. Attitudes; 3. Intended future use.

1. *Perceived enjoyment,* with a Cronbach’s α 0.94: 6 items were used to assess feelings of pleasure during exercise, e.g., “I really enjoyed exercising in the IVR environment” and participants were requested to answer through a 5-point Likert scale ranged from 1 (strongly disagree) to 5 (strongly agree) [32].
2. *Attitudes,* with a Cronbach’s α 0.89: on the basis of guidelines from theory of planned behavior, 6 bipolar items were used to assess attitudes towards VRADA app e.g., “pleasant-unpleasant”, “beautiful-ugly” and scored on a 7-point semantic differential scale [31].
3. *Intended future use,* with a Cronbach’s α 0.94: on the basis of guidelines from the theory of planned behavior, 3 items were used to assess the extent to which an individual consciously wants to use the IVR system to exercise, e.g., “assuming I have access to the system, I intend to use it”. Participants were requested to answer through a 5-point Likert scale ranged from 1 (strongly disagree) to 5 (strongly agree) [32,33].

#### 2.4.3. Usability

Subjective usability was assessed with the system usability scale (SUS), a self-report questionnaire, with a Cronbach’s α 0.71, which includes 10 items: 5 positive, e.g., “I think that I would like to use this system frequently” and 5 negative, e.g., “I found the system unnecessarily complex” [34]. Participants were requested to answer through a 5-point Likert scale ranging from 1 (strongly disagree) to 5 (strongly agree).

#### 2.4.4. Preferences

To assess the preference for the exercise with or without the IVR system, 8 questions were used e.g., “Exercise was more pleasant…”, “the numerical calculations were more fun…”—“when exercising with or without the IVR system”. Responses were dichotomous, scoring 0 (against IVR) or 1 (in favor of IVR). Respectively scoring was 0 (against the static bicycle) or 1 (in favor of the static bicycle). A composite score (total of the scores on the 8 questions) was computed for preference for the static bicycle and for preference for the IVR system. Thus, two preference scores, ranging from 0–8 were derived—One for the static bicycle and one for the IVR system.

#### 2.4.5. Interest/Enjoyment

The intrinsic motivation inventory questionnaire, with a Cronbach’s α 0.94, was used to assess the participants’ interest/enjoyment for exercise with and without the IVR system with 6 items, e.g., “I enjoyed doing this activity very much”, “This activity was fun to do”. The questionnaire was used for both training conditions. Participants were requested to answer through a 5-point Likert scale ranged from 1 (strongly disagree) to 5 (strongly agree) [35,36].

#### 2.4.6. IVR Equipment

Participants’ perceptions of using the IVR system (headset and controller) were assessed by a questionnaire with 9 questions related to usability-pleasantness, with a Cronbach’s α 0.64 (4 items e.g., “I felt comfortable using the head mounted display”), usability learning, with a Cronbach’s α 0.58 (2 items, e.g., “It was easy to read the numerical questions”) and tolerability, with a Cronbach’s α 0.38 (3 items assessing dizziness, boredom and anxiety). Participants were requested to answer through a 5-point Likert scale ranged from 1 (strongly disagree) to 5 (strongly agree) [37].

#### 2.4.7. Additional Assessments

Additional information was collected with a semi-structured interview. The semi-structured interview [38] was designed to collect qualitative data related to participants’ subjective feelings and perceptions about the reasons for using VRADA, e.g., “Why would you use VRADA training system?”, their expectations—2 items e.g., “Given the opportunity, would you be willing to use this training system regularly?”, usability or utilization—5 items, e.g., “What difficulties did you encounter during the training session?”, usability or learning, 2 items, e.g., “What exactly did you like most and least?”, sense of presence or spatial presence, 2 items, e.g., “Did you feel you had control over the environment?”, sense of presence or engagement, 2 items, e.g., “Did you get distracted during exercise? By what?”, sense of presence or realism, e.g., “How did you find the environment, realistic or too artificial?” and tolerability, 2 items, e.g., “Did you feel bad during exercise? When and where exactly?”. There were also 4 questions about the adoption of exercise with the IVR system to the participants’ working environment, e.g., “How do you think using the IVR system to exercise can help you be more productive at work?”, “If there were the VRADA system in your workplace, would you use it regularly? When? Why?”. The interview guide can be found in Supplementary Material S2.

### 2.5. Procedure

Initially, participants were informed about the procedure, completed the consent form, adjusted their position on the cycle-ergometer and they were encouraged to ask questions about the procedure. The IVR training system was easy to use and instructions given were adopted from a previous study [22]. There were two different training conditions, condition A in a virtual environment and condition B in real environment, including a combination of cognitive and physical exercise. The use of IVR technology has lately been considered as a new approach to promoting physical activity and health behavior [39]. Early studies showed that VR bike exercise condition compared to the conditions without virtual reality significantly improved attendance and adherence (83.33%) over a 14-week intervention period [40]. A recent systematic review investigated the effectiveness of exercise-based VR training in improving physical activity and performance in a healthy population compared with traditional programs. Results showed a large effect on frequency of physical activity and a small to moderate effect on physical performance [41]. Additionally, higher enjoyment and self-efficacy and lower perceived exertion were reported after a VR exercise session compared to traditional exercise [42]. Wearing the VR headset while exercising can also make people remove themselves from their bodily sensations and allow them to exercise longer [43] and improve depression symptoms [44]. The VRADA exercise simulates exercise in a natural environment making it more attractive for short bouts of exercise during working time [45].

We compared the two conditions (A and B) to investigate which one is more preferable, enjoyable and interesting. Based on previous studies that compared similar exercise conditions, we expected that condition A would be preferable for office workers as well. Several previous studies compared exercise sessions with and without VR, for example, virtual reality rehabilitation versus conventional physical therapy in Parkinson’s disease patients [46], VR cardiac rehabilitation program versus booklet or usual care [47], VR biking versus exergaming and traditional biking in college students [42] and VR biking versus traditional biking in college students [48]. Therefore, we considered it as important to follow a similar design for our comparator groups to provide richer content for our target group of office workers.

**Figure 3:**
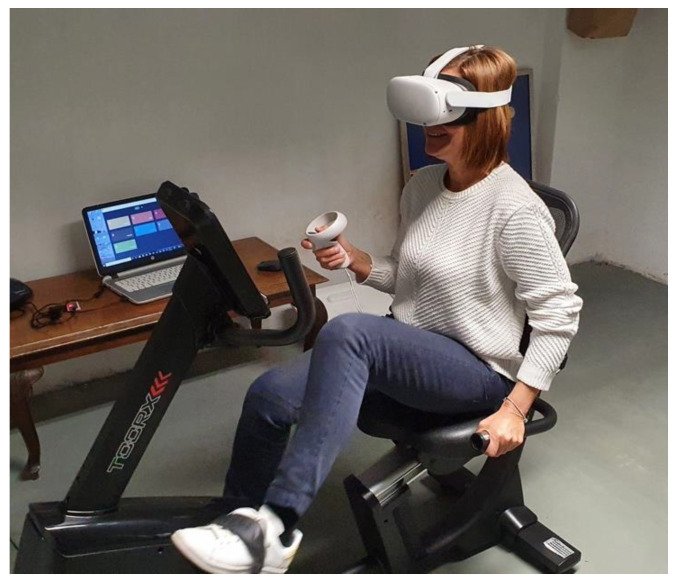
Participant performing an experiment.

Participants completed each session in counterbalanced order. For the first two minutes, each individual had time to familiarize themselves with the cycle-ergometer, the headset and the controller and then they were requested to perform condition A: to cycle for 15 min in a virtual reality environment, using the VRADA system and answer 20 simple math calculations, displayed at the screen, using the controller. They could receive feedback about the cycling time, their speed, distance and correct answers on cognitive exercises on their screen. After completing this process, they had a 10-min break and then they started condition B: to cycle for 15 min in a real environment and answer 20 numerical questions orally. During the cycling process, each subject could receive feedback about the cycling time, their speed and distance on a connected computer and at the end they were informed about their score in cognitive questions. The cycling performance was at a constant speed ranged from 15 to 20 km/h. In every trial, the bike workload and speed were adjusted according to the exercise protocol, which was the same for both conditions. (Figure 3) Finally, after a 5-min rest, they completed a questionnaire and discussed the interview questions with the experimenter. The duration of the procedure was approximately 60 min.

### 2.6. Data Analysis

Quantitative data were analyzed using SPSS Statistics version 26 (IBM Corporation). Summary statistics were calculated for demographic characteristics and correlations among all examined variables were assessed using the Pearson coefficient. To examine the preference among exercise conditions, and identify statistical differences of interest/enjoyment between the two exercise conditions, paired-samples t-test was performed. Data of the semi-structured interview were analyzed using thematic analysis [49], which can offer rich insights into attitudes and beliefs by identifying patterns of ideas and responses. The topics of the discussion were based on previous studies, the main themes were predetermined (deductive approach). Second-order themes were analyzed using an inductive approach, allowing the data to determine subthemes.

## 3. Results

Table 2 presents descriptive statistics including the means, Cronbach’s α and Pearson correlations for all the examined variables, whereas representation of data with boxplots are displayed in Supplementary Material S3.

### 3.1. Personal Innovativeness, SUS and Acceptance

The participants scored moderately to high on personal innovativeness and the SUS score was well above the acceptability threshold. Acceptance was assessed by three sub-factors: perceived enjoyment, attitudes and intended future use. Participants scored highly on all three factors: perceived enjoyment, attitudes and intended future use.

### 3.2. Relationships between Variables

Personal innovativeness was low related to interest/enjoyment without IVR variable. Perceived enjoyment was moderately related to usability, preference for condition A and usability-pleasantness, strongly with intended future use, attitudes and interest/enjoyment for condition A and moderately negative to preference for condition B. Intended future use was moderately related to interest/enjoyment for condition A, strongly positive to attitudes and preference for condition A and strongly negative to preference for condition B. Usability had low relation with attitudes, moderate with interest/enjoyment for condition A and usability-learning variable. Preference for condition A had a low negative relation with tolerability a moderate positive relation with interest/enjoyment for condition A and a strong positive relation with attitudes. Attitudes is strongly related with preference for the condition A and interest/enjoyment for the condition A, has low relation with usability-pleasantness and moderately negative related to preference for the condition B. The interest/enjoyment variable has moderate negative relation with preference for the condition B. Finally, the usability-pleasantness variable is moderately related to interest/enjoyment for condition A and usability-learning and strongly with usability.

### 3.3. Preferences and Interest/Enjoyment

A paired-samples t-test was conducted to compare the participants’ preference between condition A and condition B. There was a significant difference in the scores favoring condition A (M = 6.03, SD = 2.29) in comparison to condition B (M = 1.90, SD = 2.30); t(39) = 5.81, *p* < 0.001.

A paired-samples t-test was performed to determine whether there was a statistically significant interest/enjoyment for either of the exercising conditions. There was a significant difference in the scores favoring condition A (M = 4.21, SD = 0.78) in comparison to condition B (M = 2.86, SD = 0.86); t(39) = 7.43, *p* < 0.001.

### 3.4. Semi-Structured Interview

The collected data from the semi-structured interview related to the participants’ subjective feelings, expectations and perceptions about the VRADA’s system usability and tolerability are presented in Table 3. A great percentage of participants reported that they would use this IVR system to exercise and that they did not encounter any difficulties. Most of them did not have any problems with the equipment (headset and joystick) but more than half of the participants noted a difficulty to aim the correct answers with the joystick. None of them needed extra help or time to understand the IVR system and most of them had feelings of control and presence in the IVR environment. Concerning the use of the IVR system in the workplace many participants reported that the system may have advantages for the employees like that it can help them to relax, to be more physically active and improve their mood and wellness. Finally, many of the participants reported that they find it realistic to exercise in their workplace with the VRADA system and the majority reported that they could use it systematically. However, some of the participants noted that they do not find it realistic to exercise with the IVR system during their working hours in the workplace. Most of them supported their answer by saying that they are working in high pressure and they do not have enough time for an active break.

## 4. Discussion

The aim of this study was to examine the applicability of an immersive virtual reality system for exercise training by office workers during breaks within their working hours. We expected that participants will prefer and enjoy the IVR training condition compared to the bike only condition. The novelties of this study are new data for the acceptance, usability, future adoption and interest/enjoyment of office workers towards the IVR training system during their working hours. The acceptance of a new technology is mediated by various factors related to the user’s psychology, the design of the system and the quality of the technology [50]. In addition, the effects of exercise in a virtual reality environment depends on the feeling of presence, the task and the IVR system [51].

The constructs used for this study are based on the concept of applicability. Preference is linked to an individual’s affective response to exercise and is positively associated with exercise frequency, habit, vitality, enjoyment and wellbeing variables [52,53]. Therefore, exercise programs should be adjusted to the individual’s preference in order to lead to increased levels of adherence. Enjoyment is considered an important physical activity determinant, since it reflects feelings of pleasure, liking and satisfaction and has shown positive associations with intention, increased attendance and adherence to the exercise program [52,54]. According to our results, participants reported higher enjoyment during IVR exercise compared to regular biking. Furthermore, physical activity is associated with age, sex, self-efficacy, health status and motivation [55]. Intrinsic motivation is defined as a form of motivation deriving from the innate needs for competence and self-determination, which, when satisfied, typically result in positive feelings of control [56]. IVR cycling can promote greater intrinsic motivation compared with traditional cycling, as our results suggest and this may motivate future physical activity participation [57]. Additionally, the link between task variety and enjoyment can lead to increased intrinsic motivation [58] and VR provides greater task variety than regular stationary biking. Interest is a form of motivation characterized by a focus on a certain object [56] and it is based on the appraisals that something is new, complex, uncertain and unexpected [58], such as while exercising with IVR technology. Attitude is a strong predictor of intention to participate and adhere to physical activity [59]; therefore, attitudes towards IVR exercise and personal innovativeness have a significant effect on the adoption of new technology [60]. Nevertheless, usability is a crucial factor that must be measured and conditions that hinder someone’s interaction with the device must be taken into consideration [61]. Moreover, the greater the feeling of presence in the virtual reality that participants experience, the greater level of motivation they have [57]. All the above constructs are considered important contributing variables to assessing the applicability of the IVR exercise system that may facilitate office workers to exercise during their break of working time.

In this study, participants provided moderate to high scores on personal innovativeness, implying that they were familiarized with new technologies. They also indicated good acceptance of the IVR system as denoted by the relatively high scores in the three acceptance factors and in two usability facets. The high levels of usability indicated great acceptance and ease of use. As we hypothesized, a greater preference to condition A and high scores on interest/enjoyment in favor of IVR cycling have also been found. According to current literature, few studies compared VR exercise sessions with traditional exercise sessions and assessed preference and interest/enjoyment in various populations. The results for older people, university students and older people with mild cognitive impairment were very encouraging in favor of IVR exercise compared with traditional exercise [22,62]. Correlation results showed a positive and strong relationship between perceived enjoyment, intention for future use, attitudes and interest/enjoyment for the IVR training system. According to motivation theorists, when individuals perceive exercise as a pleasant activity, there are also positive associations with enjoyment, increased motivation, intention to continue exercise and persistence [63].

According to the results of the semi-structured interview, the majority of the participants reported that they preferred the VRADA exercising system in comparison to condition B. There were not many difficulties reported, except for the sensitivity of the joystick. All participants affirmed that they did not need extra help or time to understand the system and many of them reported that they would use it for exercising systematically. Additionally, no adverse effects were reported through the relevant items and semi-structured interview questions, for example for dizziness, nausea or discomfort, so the cybersickness effect was not detected in this study. Concerning the use of the IVR system in the workplace, although most of the participants noted that they found it realistic to use the system frequently during their working hours, there was a notable percentage reported that they did not find it realistic for many reasons, such as occupational stress and lack of time. In Greece, occupational stress is a frequent phenomenon because of economic instability. Most companies or organizations in the private and public sector are trying to manage this changing situation resulting in stressful conditions for employees [64]. Stress and quality of life are factors affected by Greece’s economic crisis and high stress levels are related to low job satisfaction and quality of life [65]. In turn, this limits the possibility for scientifically proven interventions to be implemented in the workplace [64]. However, in the European Union (EU), many organizations are preparing and trying worksite health and wellness strategies to promote employees’ health and prevent diseases. The concept of occupational safety and health differs across the EU countries, but it consists of some common elements such as healthy eating, smoking abstinence, mental health promotion, increased exercise and physical activity and health monitoring. According to the occupational safety and health campaign, a workforce that is healthy, motivated and well-qualified can become a key pillar of a healthy society with economic wellbeing [66].

Participants also reported many advantages of the IVR training system to the employees’ productivity. Employees’ productivity depends, among others, on mental health, physical health, job characteristics and support from the organization and colleagues [67]. Except for job characteristics, all of them can have positive effects from exercise in the workplace. According to a study, possible benefits can be offered for employees’ productivity and absenteeism, from a workplace exercise intervention, by improving their musculoskeletal disorders and physical health [68]. Additionally, mental health can be improved from an exercise program in the workplace [69] as well as the personal relationships between employees [6]. The VRADA system has also been tested with different groups of adults with reported high acceptability, usability and tolerability indexes by students and older people with mild cognitive impairment [22]. All things considered, we can safely claim that the IVR app had a great applicability in this population, in this Greek city, in their working environment.

### Strengths and Limitations

To the best of our knowledge, no previous study has examined the perceptions of office workers for an IVR exercise system using a static bicycle during working hours. The VRADA system includes motivational techniques to address the issue of low motivation to exercise, for example, goal setting and feedback. Additionally, the users have the opportunity to choose their pace, velocity, landscape and music. The system can be adjusted to individual needs and installed in the workplace to be easily accessible for the employees.

Despite these encouraging results about the acceptance, usability, interest/enjoyment and intention for future use, there are some limitations. Since the VRADA system has been tested for one session, long-term interventions have to be tested to examine if this way of training will remain enjoyable and interesting, as well as the training effect [22,70]. Moreover, future research could include assessments of exercise intensity using this application. Moderate intensity exercise is commonly considered beneficial for health, but we suggest a “self-selected intensity” plan. According to current research, when people select the exercise intensity by themselves, the sense of autonomy is promoted and that is related with more adaptive motivational and behavioral outcomes in a physical activity setting [71]. Specially, the frequency and intensity of IVR exercise programs during working hours can be increased to be more challenging for employees, but duration must remain low in order to be realistic as an option for exercise during working hours. Nevertheless, there were no adverse effects in this single 15-min session, but in long-term implementation, the exercise duration and frequency must be taken into consideration. According to recent literature, low duration and frequency of IVR exercise minimize the cybersickness effect [72]. We assessed cybersickness effects with three items included in the questionnaire and semi-structured interview. We suggest future studies should use the simulator sickness questionnaire (SSQ) so the data could be more comparable with other studies. According to a systematic review and meta-analysis, which investigated SSQ scores related with VR exercise in multiple studies, pooled participants’ scores were relatively high and content was considered as a major contributing factor [73]. Additionally, the sample size was small and participants were women only, because more women volunteered to participate in this study and the offices where we addressed were female-dominated. Therefore, the emerged results cannot be generalized to the general population because male participants were not included. There is a need for more studies to examine the IVR system training in the workplace during working hours with a larger sample and gender balance. The aim of this study was to examine the applicability of the VRADA system, and not the efficacy and users’ adherence in the exercise program. Future studies could evaluate the effects of regular training with the VRADA system on physical and mental health and quality of life.

## 5. Conclusions

Overall, exercising during working hours with an IVR exercise system was well perceived by office workers and applicable. Nevertheless, long-term intervention studies are required to explore the effectiveness on long-term benefits for several health and fitness dimensions and cost effectiveness of IVR exercise in sedentary workplace settings.

## Figures and Tables

**Table 1:** Educational level, habits of physical activity and technology use.

Baseline Characteristic	*n*	%
Educational Level		
Secondary education	5	12.5%
Higher education	35	87.5%
Exercise program		
Yes	17	42.5%
No	23	57.5%
Physical Activity: Times Per Week		
One time per week	2	5%
Two times per week	4	10%
Three times per week	7	175%
Four times per week	2	5%
Five times per week	6	15%
Six Times Per Week	2	5%
Physical Activity: Hours Per Time		
45 min	5	12.5%
1 h	14	35%
1.5 h	4	10%
Phone Use		
Never	1	2.5%
1–2 h per day	17	42.5%
3–4 h per day	16	40%
5–6 h per day	4	10%
Up to 6 h per day	2	5%
Pc Use		
Never	2	5%
1–2 h per day	5	12.5%
3–4 h per day	4	10%
5–6 h per day	5	12.5%
Up to 6 h per day	24	60%
Video Game Use		
Never	35	87.5%
1–2 h per day	5	12.5%

**Table 2:** Main outcomes in the variables studied.

Variables	PI	PE	IFU	SUS	AT	IMI_A	IMI_B	PRE_A	PRE_B	UP	UL	MEAN	SD	Cronbach α
PI ^a^												3.55/5	0.68	0.77
PE ^b^	0.22											4.34/5	0.80	0.94
IFU ^c^	0.25	0.80 **										4.02/5	0.97	0.94
SUS ^d^	0.07	0.41 **	0.26									80.38/100	11.13	0.71
AT ^e^	0.23	0.87 **	0.87 **	0.31 *								6.06/7	1.00	0.89
IMI_A ^f^	0.27	0.83 **	0.68 **	0.42 **	0.82 **							4.21/5	0.78	0.94
IMI_B ^g^	0.40 *	−0.00	−0.06	−0.01	−0.15	0.02						2.86/5	0.85	0.94
PRE_A ^h^	0.07	0.68 **	0.72 **	0.18	0.73 **	0.64 **	−0.19					6.03/8	2.29	−
PRE_B ^i^	−0.05	−0.69 **	−0.71 **	−0.20	−0.75 **	−0.66 **	0.21	−0.99 **				1.90/8	2.30	−
UP ^j^	0.18	0.46 **	0.29	0.71 **	0.32 *	0.52 **	0.03	0.25	−0.25			4.18/5	0.56	0.64
UL ^k^	0.17	0.09	0.06	0.52 **	0.00	0.15	0.20	0.08	−0.63	0.66 **		4.25/5	0.62	0.58
TOL ^l^	−0.17	−0.21	−0.20	−0.17	−0.24	−0.36	−0.03	−0.32 *	−0.32 *	−0.31	−0.14	1.77/5	0.69	0.38

* Correlation is significant at the 0.05 level, ** Correlation is significant at the 0.01 level. ^a^ PI = personal innovativeness, ^b^ PE = perceived enjoyment, ^c^ IFU = intended future use, ^d^ SUS = system usability scale, ^e^ AT = attitudes, ^f^ IMI_A = interest/enjoyment for condition A (IVR) (intrinsic motivation inventory), ^g^ IMI_B = interest/enjoyment for condition B (intrinsic motivation inventory), ^h^ PRE_A = preference for condition A (IVR), ^i^ PRE_B = preference for condition B (bike only), ^j^ UP = usability—pleasantness, ^k^ UL = usability—learning, ^l^ TOL = tolerability.

**Table 3:** Subjective feelings, expectations and perceptions about the VRADA system, its usability and tolerability.

Main Themes	Subthemes	*n*	(%)
Reasons to use VRADA	Why would you use VRADA? Because:		
	• It is pleasant	25	62.5%
	• Time passed fast and delightfully	12	30%
	• It is interesting	7	17.5%
	• It is relaxing	6	15%
Expectations	Future personal use of the system		
	• Yes	24	60%
	• So-So	5	12.5%
	• No	11	27.5%
	Useful for other populations		
	• People who cannot exercise outdoors	12	30%
	• People who do not like to exercise, motivation	9	22.5%
	• Children and adolescents	9	22.5%
	• Disabilities	7	17.5%
	• Everybody	6	15%
	• People with limited free time	5	12.5%
	• People with mental disorders	5	12.5%
Usability or utilization	General difficulties		
	• None	27	67.5%
	• Combination of questions and searching for animals	4	10%
	• Questions placed very low	4	10%
	• To find animals	4	10%
	Technical issues		
	• To aim the correct answers with the joystick	22	55%
	• None	16	40%
	• The system was disconnected	2	5%
	Joystick use		
	• Ok	36	90%
	• Ineffective	2	5%
	• The button	1	2.5%
	IVR HMD use		
	• Ok	27	67.5%
	• Did not fit properly	9	22.5%
	• It was heavy	6	15%
Usability or learning	Need for extra help		
	• No	40	100%
	Need more time to understand the system		
	• No	40	100%
Usability or pleasantness	Most enjoyable parts		
	• The environment	32	80%
	• Music	17	42.5%
	Least enjoyable parts		
	• Repeated virtual parts	15	37.5%
	• Graphics	9	22.5%
	• Questions	8	20%
	Feel uncomfortable		
	• No	38	95%
	• I felt alone	1	2.5%
	• When I was searching for the animals	1	2.5%
Sense of presence or spatial presence	Control of the system		
	• Yes	33	82.5%
	• Not really	3	7.5%
	• Almost yes	2	5%
	• No	2	5%
	Feel part of the VR environment		
	• Yes	26	65%
	• Almost yes	9	22.5%
	• No	3	7.5%
	• Not really	2	5%
Sense of presence or engagement	Distraction of attention		
	• No	34	85%
	• Yes (e.g., when animals appeared)	6	15%
	Duration of the experience		
	• Enough	20	50%
	• It could be more	19	47.5%
	• It could be less	1	2.5%
Sense of presence or realism	VR environment was realistic or artificial		
	• Artificial	33	82.5%
	• Realistic	7	17.5%
Tolerability	Feel bad during training		
	• No	36	90%
	• Yes (anxiety, fear)	4	10%
Use in workplace	Advantages of the system for employees		
	• Help them to relax	17	42.5%
	• Help them to be physically active	10	25%
	• Improve mood and wellness	9	22.5%
	• Have a delightful break from work	8	20%
	• Strengthen physical and mental health	4	10%
	Help to be more productive		
	• Allow my mind to rest	13	32.5%
	• Calm down from stress and tension	12	30%
	• Have a delightful break from work	11	27.5%
	• It cannot help	3	7.5%
	How realistic is systematically exercise in workplace with the VR system		
	• Very realistic	16	40%
	• Not at all	10	25%
	• Not very realistic	9	22.5%
	• Quite realistic	5	12.5%
	Systematically use		
	• Yes	29	72.5%
	• No	11	27.5%

## Data Availability

Not applicable.

## Supplementary Materials

The following supporting information can be downloaded at: https://www.mdpi.com/article/10.3390/sports10070104/s1, Supplementary Material S1: Video demo; Supplementary Material S2: Interview Guide; Supplementary Material S3: Boxplots.
