# Supplementary material for: Applicability of an Immersive Virtual Reality Exercise Training System for Office Workers during Working Hours
Source: Sports (Basel). 2022 Jun 29;10(7):104. doi: 10.3390/sports10070104 (PMC9317041; doi:10.3390/sports10070104)
Supplement: Supplementary file 1 [file sports-10-00104-s001.zip › Supplementary Material S2 - Iterview Guide 2.pdf]

Interview Guide (semi-structured)

1. Why would you want to use the Virtual Reality system to exercise?
2. What difficulties did you encounter while exercising with the Virtual Reality system?
3. Were there any technical issues while exercising with the Virtual Reality system?
4. Did you have to ask for help to understand how to use the Virtual Reality system? At what point did you most need help? Do you think more instructions are needed?
5. Did it take long to figure out how the Virtual Reality system works? What took most time to figure out? Was the trial sufficient to comprehend the Virtual Reality system?
6. Was the use of the joystick difficult/complicated (e.g., buttons)? What aspect of using the joystick gave you most trouble?
7. What did you like most and what did you like least about the Virtual Reality system?
8. Was there any point at which the Virtual Reality system made you feel uncomfortable?
9. What problems (if any) did you have with the Virtual Reality mask during the stationary bike exercise?
10. Did you feel part of the virtual environment?
11. Do you feel you had control over the virtual environment?
12. What do you think about the duration of the experience?
13. Were you easily distracted while exercising with the Virtual Reality system? At what point did you get distracted?
14. How did you find the environment? Was it realistic or too artificial?
15. Did you feel uncomfortable at any moment when exercising with the Virtual Reality system?
16. Did you experience any nausea, dizziness or other physical symptoms when exercising with the Virtual Reality system?
17. Would you like to use this Virtual Reality system as part of a regular exercise regime?
18. Do you think this system would be useful for other people?
19. What advantages do you think the systematic use of the VR system by the employees in you workplace would have?
20. How do you think using the VR system to exercise can help you be more productive at work?
21. How realistic do you find it to exercise systematically with the VR system located in your workplace?
22. If there were the VRADA system in your workplace, would you use it regularly? When? Why?
