# Supplementary material for: Applicability of an Immersive Virtual Reality Exercise Training System for Office Workers during Working Hours
Source: Sports (Basel). 2022 Jun 29;10(7):104. doi: 10.3390/sports10070104 (PMC9317041; doi:10.3390/sports10070104)

Boxplots

Personal Innovativeness

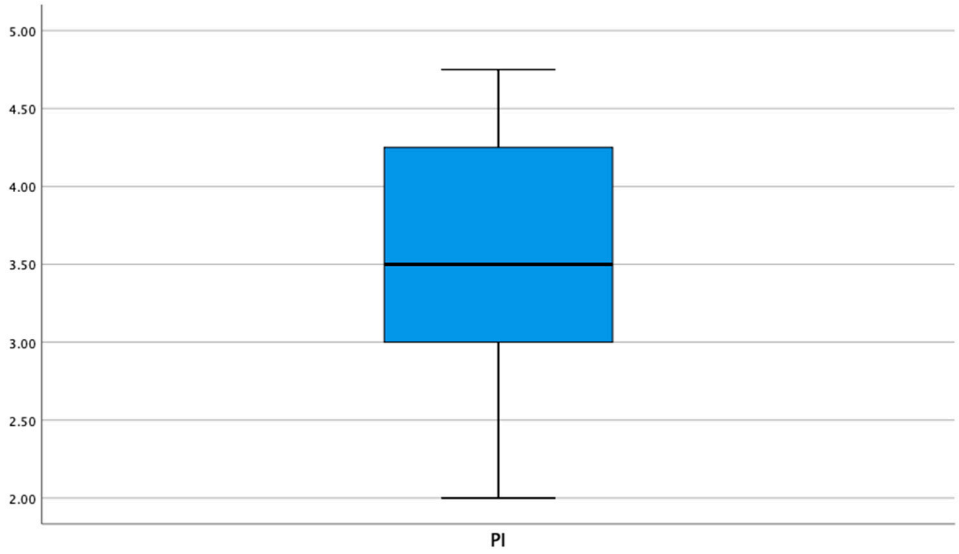

Perceived Enjoyment

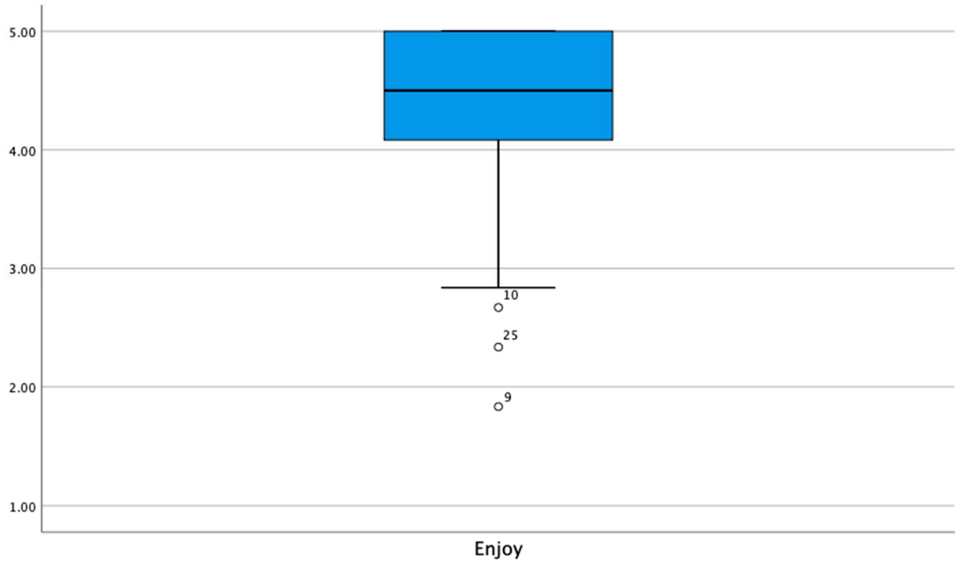

Intention

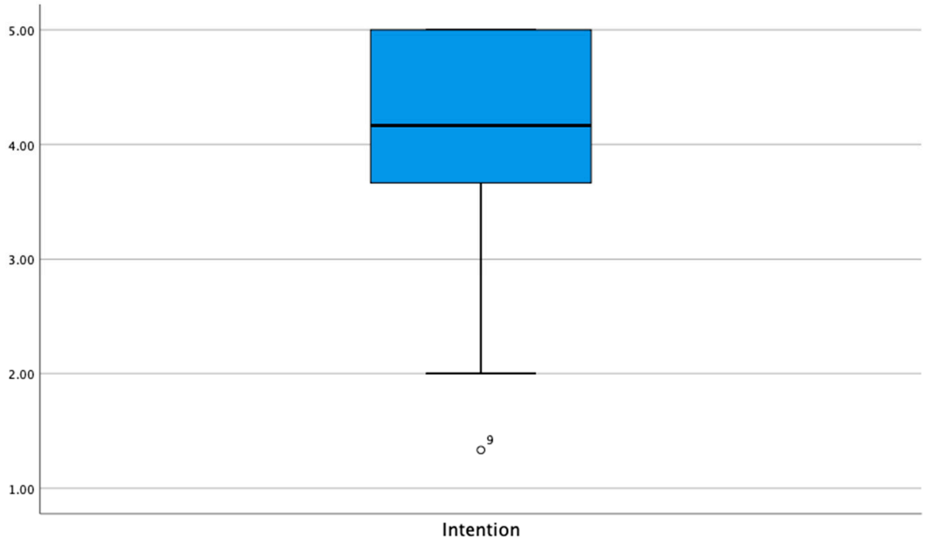

Usability

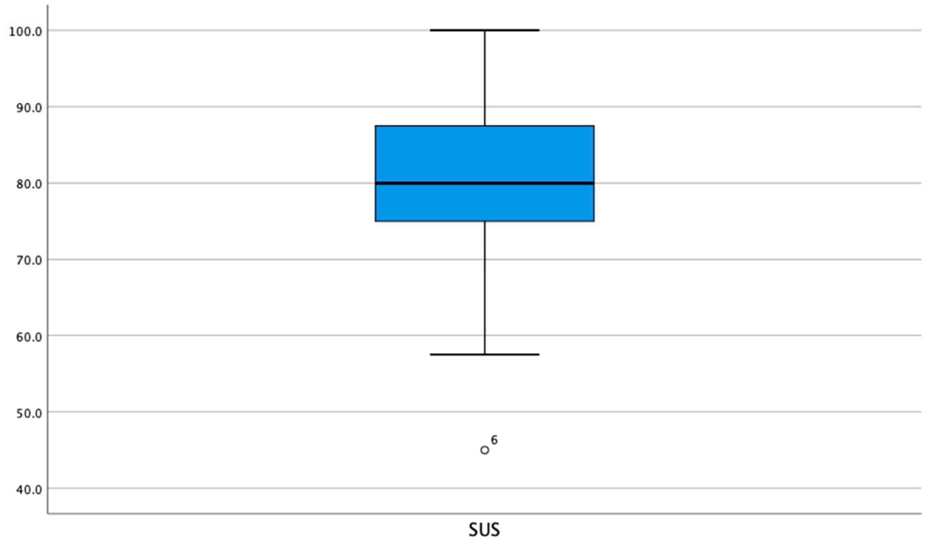

VR equipment

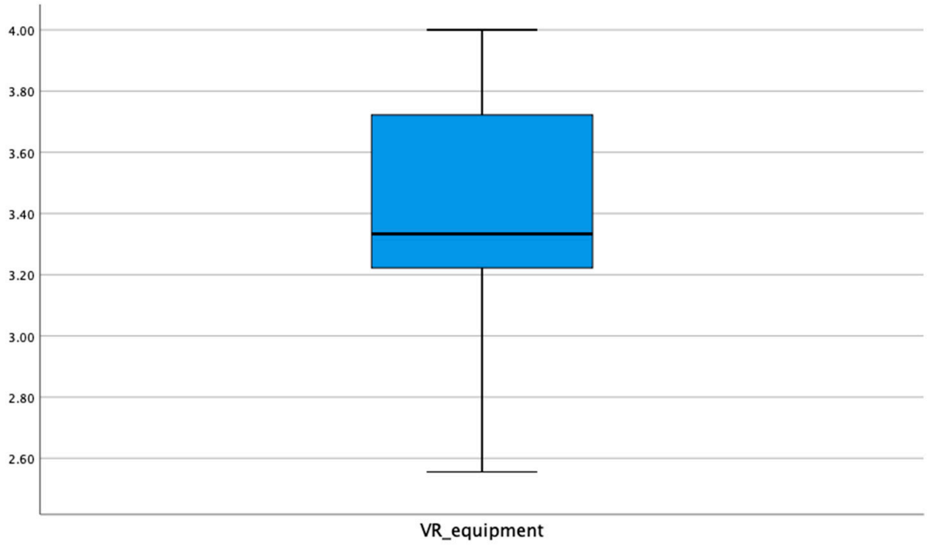
4.00
3.80
3.60
3.40
3.20
3.00
2.80
2.60
VR_equipment

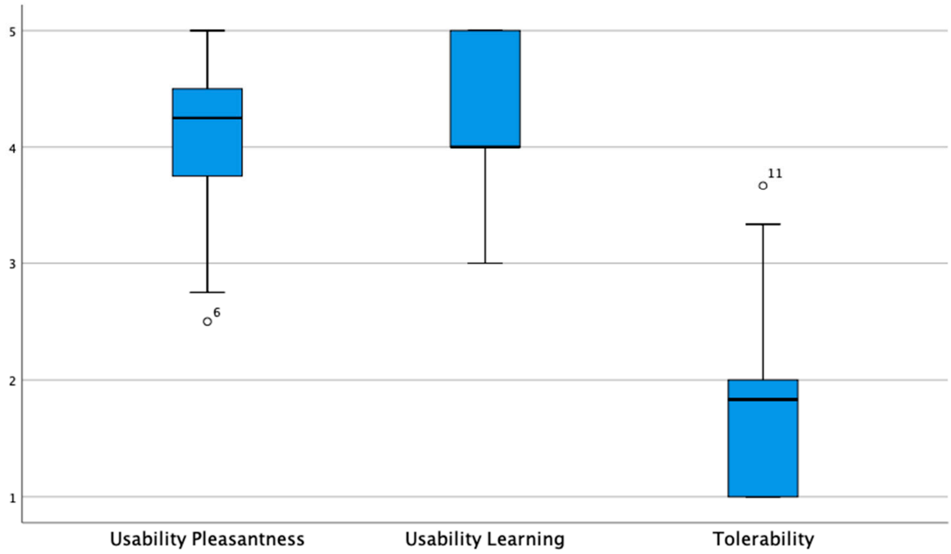
5
4
3
2
1
11
6
Usability Pleasantness
Usability Learning
Tolerability

Attitudes

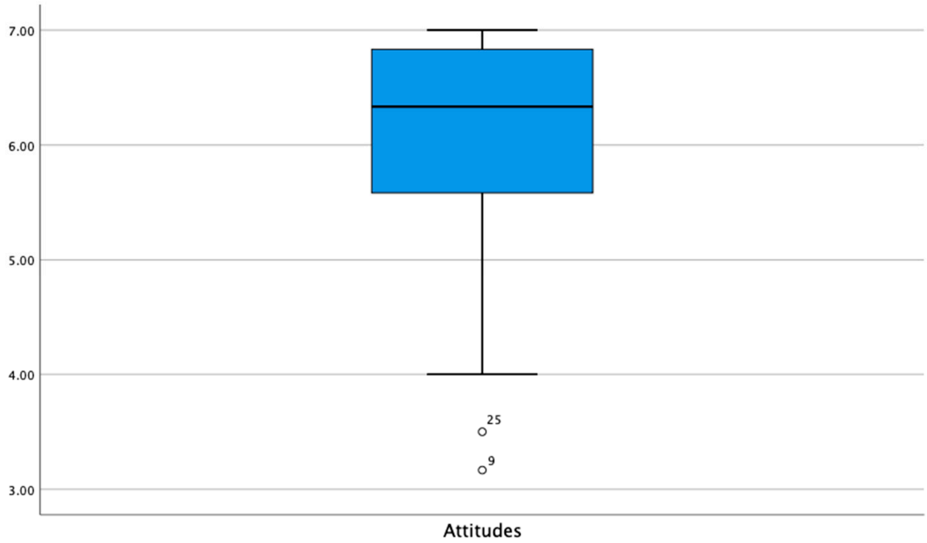
7.00
6.00
5.00
4.00
3.00
25
9
Attitudes

Interest/enjoyment

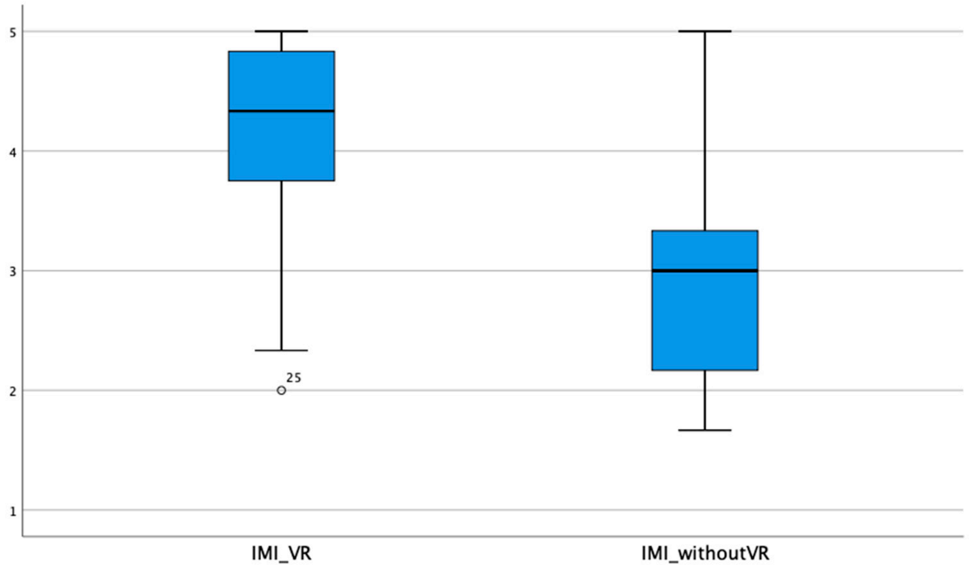

Preference

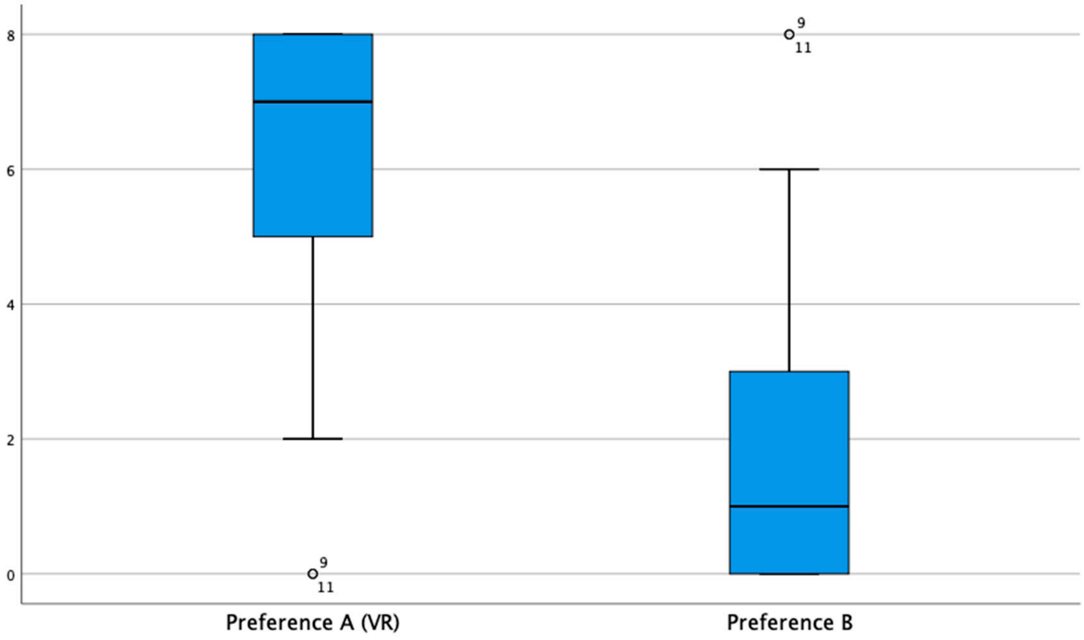

Supplement: Supplementary file 1 [file sports-10-00104-s001.zip › Supplementary material S3 - Boxplots.pdf]
